# Case Report: Proportionate short stature in a three-generation family harboring *FGFR3* N540S: phenotypic expansion beyond hypochondroplasia and implications for genetic screening in idiopathic short stature

**DOI:** 10.3389/fendo.2026.1881876

**Published:** 2026-09-03

**Authors:** Beibei Zhang, Xinmeng Wang, Jingjie Luo, Chunxiu Gong

**Affiliations:** Department of Endocrinology, Genetics and Metabolism, Beijing Children’s Hospital, Capital Medical University, National Center for Children’s Health, Beijing, China

**Keywords:** familial short stature, *FGFR3* protein, growth hormone, N540S, pedigree

## Abstract

**Objective:**

To report the identification of a *FGFR3* gene variant in a family with idiopathic short stature, characterized by proportionate stature and the absence of dysmorphic features, and to highlight the importance of genetic investigation in ISS as well as the clinical heterogeneity of the *FGFR3* N540S variant.

**Methods:**

Based on clinical and genetic data collected from the proband and short-statured relatives, we analyzed the clinical characteristics of the pedigree and the pathogenicity of the genetic variant.

**Results:**

A 3-year-old male presented with proportionate short stature and no dysmorphic features. Imaging studies revealed no significant skeletal abnormalities. Combined with the short stature observed in multiple family members, a clinical diagnosis of FSS was established. Whole-exome sequencing identified a maternally inherited heterozygous *FGFR3* variant (c.1619A>G, p.N540S) in the proband. The mother and maternal grandfather also harbored this variant. ACMG classification deemed it likely pathogenic. Literature review revealed that all previously reported patients with the *FGFR3* N540S variant exhibited clinical manifestations of HCH with abnormal radiographic features.

**Conclusion:**

This is the first report of proportionate short stature associated with the *FGFR3* N540S variant, demonstrating substantial phenotypic heterogeneity at this site. Genetic testing is strongly recommended for patients with FSS, particularly those with severe short stature.

## Introduction

Idiopathic short stature (ISS) represents a heterogeneous group of disorders characterized by short stature of temporarily unidentified etiology, clinically manifesting as reduced linear growth without significant phenotypic abnormalities. Familial short stature (FSS) constitutes a recognized subtype of ISS. FSS is defined as a height of more than 2 standard deviations (SD) below the mean for age and sex, with the height being appropriate for the mid-parental target height (1). Genetic testing in patients diagnosed with ISS/FSS has historically identified various underlying genetic conditions, including the Laron syndrome, achondroplasia, acromicric dysplasia, Silver-Russell syndrome, IMAGe syndrome, and Prader-Willi syndrome. Subsequent comprehensive clinical evaluation often reveals subtle, previously unrecognized dysmorphic features or radiographic abnormalities, ultimately establishing a diagnosis of genetic short stature. Consequently, the 2023 Chinese Expert Consensus encourages genetic testing in ISS patients, with strong recommendation for those with height SD below −3 SD or FSS (2).

Fibroblast growth factor receptor 3 (FGFR3), a member of the tyrosine kinase receptor family, encodes a receptor protein that primarily binds fibroblast growth factors and modulates their signaling pathways, playing critical roles in embryonic development, skeletal growth, and tissue repair (3). FGFR3 is predominantly expressed in the human skeleton and acts as a key physiological negative regulator via suppressing chondrocyte proliferation. Gain-of-function variants in *FGFR3* have been associated with skeletal dysplasias, predominantly manifesting as short stature, characteristic facies, and disproportionate limb shortening. Variants in different regions of *FGFR3* lead to distinct skeletal dysplasias, most of which exhibit an autosomal dominant inheritance pattern, including achondroplasia (ACH), hypochondroplasia (HCH), thanatophoric dysplasia (TD), and severe achondroplasia with developmental delay and acanthosis nigricans (SADDAN) (4).

Commonly reported pathogenic variants of *FGFR3* include: p.G380R (c.1138G>A/C), predominantly linked to ACH; p.N540K (c.1620C>A/G), accounting for approximately 70% of HCH cases; p.K650M (c.1949A>T), which primarily causes SADDAN; and p.R248C, p.Y373C, p.K650E, p.X807G, p.X807R, and p.X807C, all of which are predominantly associated with TD (5). N540-associated HCH characteristically demonstrates attenuated phenotypic severity in infancy, with clinical manifestations-including disproportionate short stature with rhizomelic predominance, lumbar hyperlordosis, acromesomelic limb shortening, and macrocephaly, typically emerging beyond 24 months of age. With advancing age, rhizomelic shortening of the lower extremities progresses disproportionately compared with the upper limbs (5). Radiographic hallmarks include progressive interpediculate narrowing from L1 to L5, anteroposterior vertebral body constriction, iliac hypoplasia, tubular bone shortening, femoral neck broadening with concomitant shortening, and metaphyseal flaring of long bones (6). Deficient infantile and pubertal growth spurts in HCH culminate in severely attenuated final adult height (mean ± SD: 143.6–148 cm in males; 130.8–139 cm in females) (7).

Among *FGFR3* variants reported in FSS cohorts, most have been associated with disproportionate short stature (8). To date, only two pedigrees, encompassing eight individuals, have exhibited proportionate short stature, with the causative variants identified as p.M528I (c.1584G>T) and p.F384L (c.1150T>C) (9). The p.N540S variant has been established as pathogenic or likely pathogenic; however, all reported cases have uniformly manifested HCH (8, 10–13). Herein, we describe a pedigree with proportionate short stature attributable to the N540S variant, in the absence of HCH features, and elucidate the mechanisms underlying this atypical phenotypic expression.

## Case report

### Patient and family

The proband was a 3-year-old male, the first child of non-consanguineous parents. The pregnancy course was unremarkable. Because maternal short stature precluded vaginal delivery, he was born via cesarean section at 38 weeks’ gestation. Birth weight was 2.9 kg (−1.19 SD), and birth length was 48 cm (−1.43 SD). No perinatal asphyxia or resuscitation was required. Feeding was uneventful, and gross motor and intellectual development proceeded within normal limits. During the first year of life, linear growth was approximately 22 cm, with achieved height of 70 cm (−2.48 SD); weight was not monitored. The patient presented for evaluation owing to persistent short stature since birth and progressive height deceleration. The mother reported substantial distress related to the psychosocial consequences of short stature and wished to initiate early intervention.

**Figure 1:**
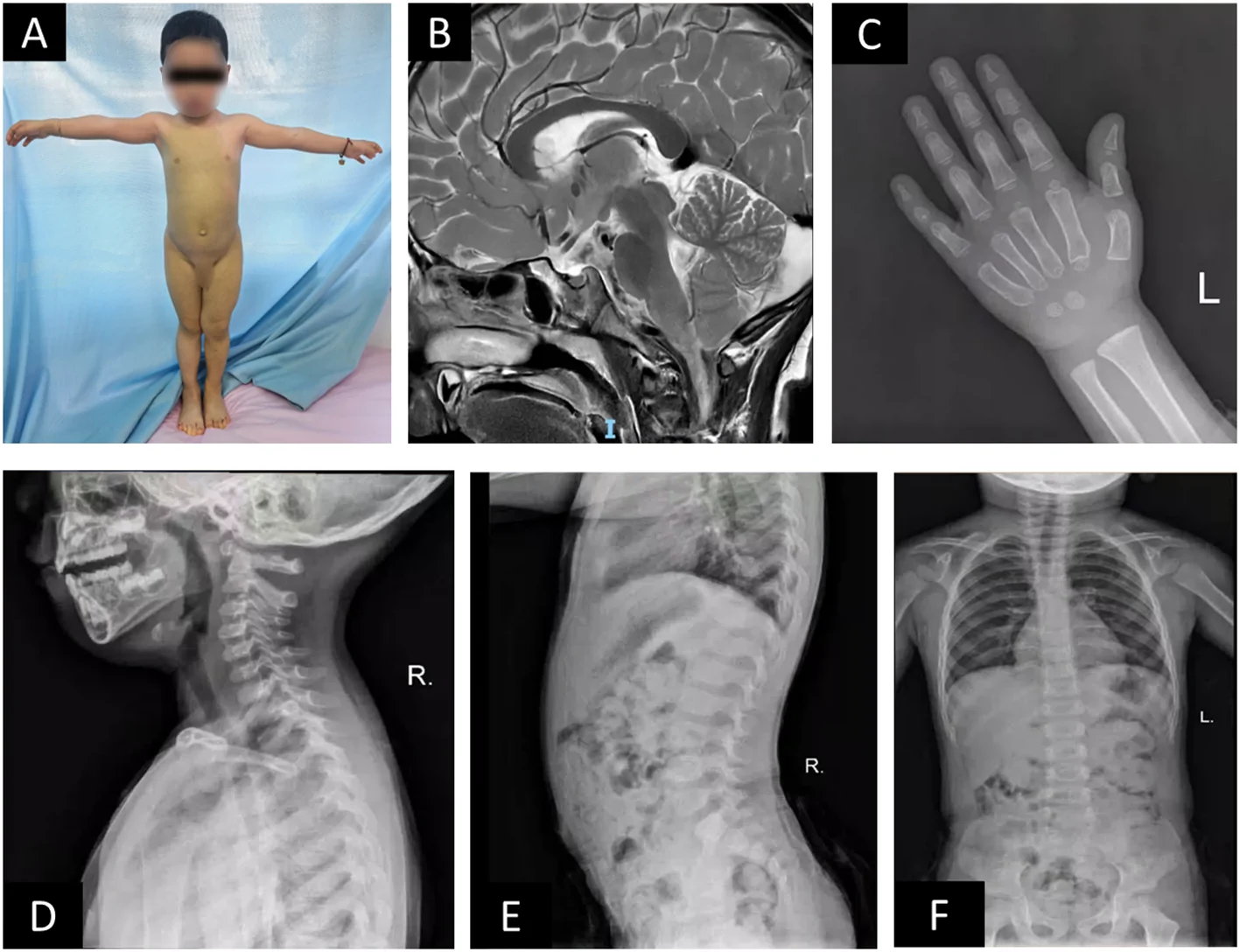
Clinical and radiographic features of the proband. **(A)**Photograph demonstrating proportionate short stature. **(B)** Pituitary magnetic resonance imaging (MRI) showing no structural abnormalities. **(C)** Bone age assessment consistent with chronological age. **(D–F)** Full-spine radiographs showing mild scoliosis without definitive vertebral malformations.

On examination, his height was 89 cm (−2.1 SD) and his weight was 13.8 kg (−1.32 SD). The patient was alert and cooperative, with coherent responses, normal gait, and unremarkable facies. Body habitus was proportionate, with an arm span of 87 cm and an upper-to-lower segment ratio of 1.25:1. No rhizomelic limb shortening was evident. Cardiopulmonary and abdominal examinations were unremarkable (**Figure 1A**).

Family history investigation revealed predominantly short stature among maternal relatives. The 32-year-old mother measured 134 cm (−4.91 SD), the 60-year-old maternal grandmother was 140 cm (−3.8 SD), and the 61-year-old maternal grandfather was 148 cm (−4.06 SD). The father measured 170 cm (−0.44 SD), the paternal grandmother 155 cm (−1.04 SD), and the paternal grandfather 170 cm (−0.44 SD).

The mother’s growth trajectory was notable for term vaginal delivery (birth weight 3.0 kg, birth length unknown) and lifelong proportionate short stature with normal motor and cognitive development. Menarche occurred at age 10 years, at which time her height had already approximated final adult stature (~130 cm). No medical intervention was pursued due to limited healthcare access at that time. The father’s perinatal and postnatal growth and development were unremarkable. There is no consanguinity between the proband’s maternal grandparents and paternal grandparents. Historical growth data for the grandparents were unavailable. All four individuals had normal ambulation.

Extended pedigree analysis of the maternal grandfather revealed nine siblings. Excluding the maternal grandfather himself, all male siblings exceeded 160 cm in height, and all female siblings exceeded 150 cm. No short stature was observed among their descendants.

### Laboratory and imaging investigations

Comprehensive laboratory evaluation revealed normal complete blood count, serum electrolytes, hepatic and renal function panels, thyroid function tests, 8:00 AM cortisol, and 8:00 AM adrenocorticotropic hormone. Insulin-like growth factor-1 (IGF-1) was 112 ng/mL (+0.37SD) (14). Karyotype analysis demonstrated a 46,XY male karyotype. Pituitary magnetic resonance imaging (MRI) and abdominal ultrasonography were unremarkable. Full-spine radiography was evaluated by a pediatric radiologist experienced in skeletal dysplasia imaging and showed no definitive vertebral anomalies. Bone age assessment was concordant with chronological age (~3 years) (**Figures 1B–F**).

### Genetic analysis

**Figure 2:**
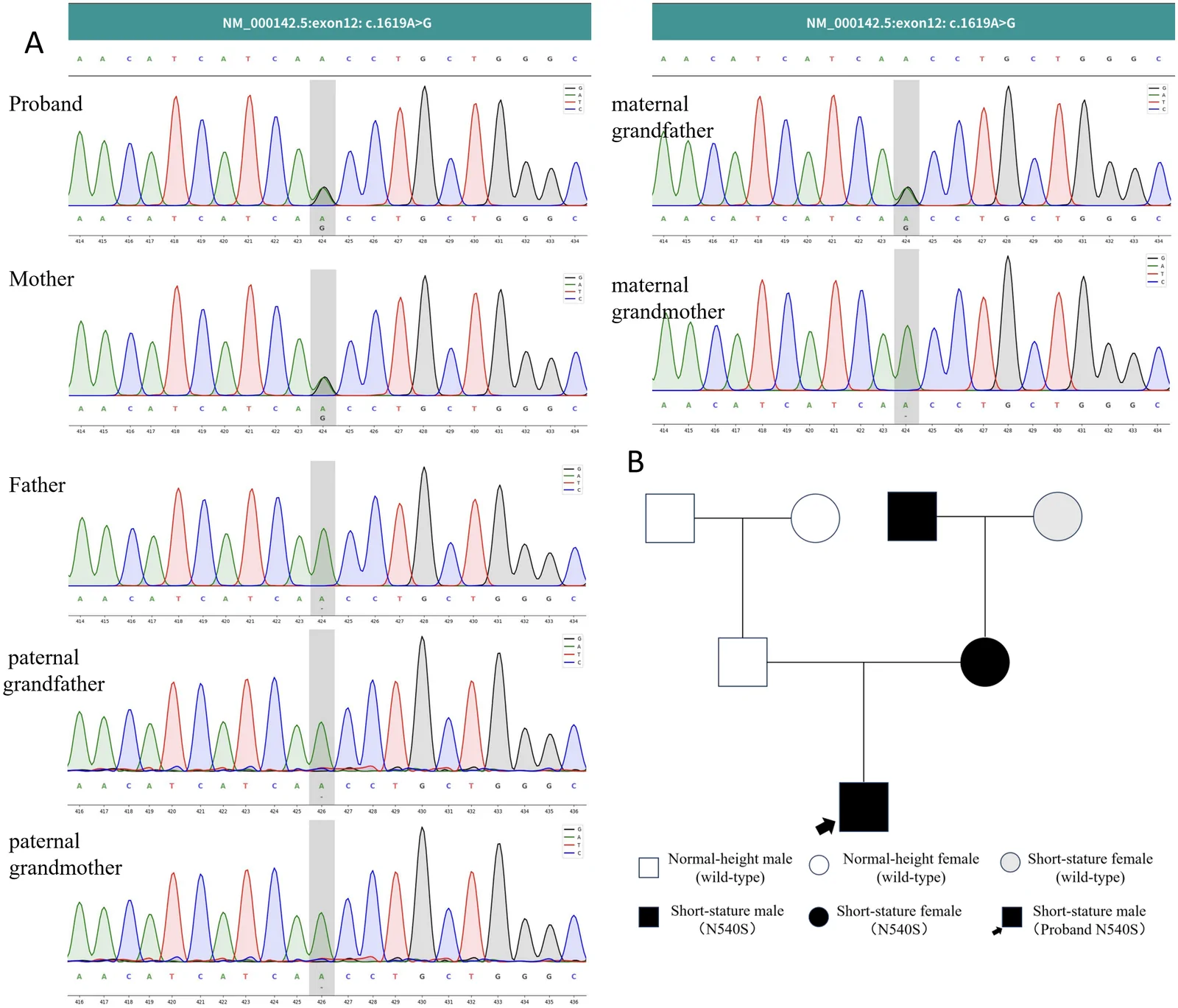
*FGFR3* variant locus and pedigree. **(A)** Sanger sequencing chromatograms showing the heterozygous c.1619A>G (p.Asn540Ser) variant in the proband, the mother and the maternal grandfather, and wild-type sequence in the father, paternal grandfather, paternal grandmother, and maternal grandmother. **(B)** Pedigree of the family. The proband (III-1), his father (II-1), mother (II-2), paternal grandfather (I-1), paternal grandmother (I-2), maternal grandfather (I-3), and maternal grandmother (I-4) are shown. Solid symbols denote affected individuals; open symbols denote unaffected individuals. The arrow indicates the proband.

Trio-based whole-exome sequencing identified a heterozygous missense variant in *FGFR3* (NM_000142.5): c.1619A>G (p.N540S) in the proband, confirmed to be maternally inherited. Sanger validation demonstrated that the maternal grandfather was heterozygous for this variant, whereas the father, paternal grandfather, paternal grandmother, and maternal grandmother were wild-type (**Figure 2**). This variant was classified as likely pathogenic by ACMG guidelines (PS1 + PM2 + PP1 + PP4).

### Patient’s follow-up

Following definitive diagnosis, recombinant human growth hormone (rhGH) therapy was initiated in the patient. At the most recent follow-up (9 months of treatment), his height was 97 cm (−0.92 SD) and his weight was 16.4 kg (+0.32SD). Biochemical surveillance, including thyroid function tests and glycated hemoglobin, remained within normal limits. IGF-1 was 124 ng/mL (+0.49SD). The patient remains under active follow-up.

### Review of published *FGFR3* N540S variants

Previously reported cases harboring the *FGFR3* N540S variant are summarized in **Table 1**. With the exception of the three probands presented herein, who were classified as proportionate familial short stature (FSS), all other reported individuals received a diagnosis of HCH. The median age at diagnosis was 9.45 years (range: 2 months to 61 years), with marked male predominance (male:female = 11:3). Variable severity of short stature was observed, including some patients with height within the normal range. Radiographic skeletal abnormalities of heterogeneous patterns were documented.

**Table 1:** Characteristics of previously reported FGFR3 N540S variants.

Patient source	Sex	Age at diagnosis (y)	Diagnosis	Height (SDS/Percentile)	Clinical features	Radiographic findings
Fang D et al., 2024 (8)	Male	4.67	HCH	-4.67SD	Facial dysmorphism, Skeletal abnormalities	Not described
De Sanctis V et al., 2012 (10)	Male (proband)	8.9	HCH	-2.2SD	Disproportionate short stature, slight bowlegs and stocky hands	Moderate narrowing of interpedicular distances between lumbar vertebrae L1 and L5, anteroposterior shortening of pedicles and increased dorsal concavity of the vertebral bodies in the lumbar spine, short and broad femoral neck,elongated distal end of the fibula with respect to tibia and mild shortening of 4th and 5th metacarpal bones.
	Male (father)	31	HCH	-1.6SD	Short stature and bowlegs	No radiographic data available
Riepe FG et al.2005 (11)	Female (proband)	13	HCH	-2.6SD	Disproportionate short stature	No characteristic radiographic features
	Male (brother)	10	HCH	-1.4SD	Disproportionate short stature, relatively large head	No characteristic radiographic features
	Male (brother)	6	HCH	-2SD	Disproportionate short stature	No radiographic data available
Thauvin-Robinet C et al., 2003 (12)	Male (proband)	0.17	HCH	Normal height	Slightly large head, short limbs	Mild lumbar kyphosis, normal interpediculate distances from the first to the fifth vertebral body, mild cuboid-shaped defect of the vertebral bodies, and short tubular bones with broad femoral necks
	Male (brother)	2.5	HCH	Normal height	Macrocephaly with frontal bossing, mild micromelia	Slightly horizontal acetabular roofs, mild metaphyseal flaring, short metacarpals
	Male (father)	32	HCH	Normal height	Micromelia, micromelia	Macrocephaly with large frontal sinuses, concave vertebral bodies with short pedicles, short iliac bones, short broad long bones and femoral heads, rectangular proximal tibial epiphysis, normal fibula, and small hands with short metacarpals.
Mortier G et al., 2000 (13)	Female (proband)	8	HCH	<P3	Mild disproportionate short stature, prominent forehead, low nasal bridge, anteroposteriorly flattened thorax, lumbar hyperlordosis	Mild shortening of the tubular bones, minimal increase in lumbar interpedicular distance, anteroposterior shortening of the lumbar pedicles and vertebral bodies, accentuated lumbar lordosis with horizontally tilted sacrum
	Male (father)	37	HCH	P3-P25	Macrocephaly with a prominent forehead, low nasal bridge, muscular build, broad thorax	Mild shortening of the tubular bones, increase in lumbar interpedicular distance, anteroposterior shortening of the lumbar pedicles and vertebral bodies, long proximal portion of the fibula, remarkably short femoral necks
The study	Male (proband)	3	FSS	-2.1SD	Proportionate short stature	Full-spine radiograph revealed mild scoliosis without accompanying vertebral anomalies
	Female (mother)	32	FSS	-4.91SD	Proportionate short stature	No radiographic data available
	Male (maternal grandfather)	61	FSS	-4.06SD	Proportionate short stature	No radiographic data available

## Discussion

ISS represents a heterogeneous group of growth disorders with unidentified etiology but diverse underlying causes. The Chinese clinical practice guidelines recommend genetic testing for ISS, particularly in children with severe short stature (2). Although the proband in this study did not meet the criteria for severe short stature at presentation given his young age, the striking familial aggregation of extreme short stature—manifested in the mother (134 cm), maternal grandfather, and maternal grandmother—coupled with the patient’s failure to demonstrate adequate catch-up growth, which fulfilled the indications for genetic evaluation. Trio-based whole-exome sequencing subsequently identified the *FGFR3* variant segregating within this pedigree. Gain-of-function variants at the N540 residue of FGFR3 constitute the principal molecular etiology of HCH, with c.1620C>A and c.1620C>G (p.N540K) being the most prevalent alterations. HCH lacks established diagnostic criteria and requires the integration of clinical, radiographic, and genetic findings. Its diagnosis remains challenging due to phenotypic overlap with other skeletal dysplasias (6).

The N540S variant, located at the same codon as N540K, has been uniformly associated with HCH in prior reports, characterized by disproportionate short stature, distinctive facial features, and skeletal malformations. This study reports the first pedigree harboring *FGFR3* N540S without somatic disproportion or skeletal radiographic abnormalities. The proband, mother (32 years), and maternal grandfather (61 years) exhibited isolated proportionate short stature without dysmorphic features, with no emergence of facial or skeletal anomalies over time. Collectively, these three cases support a diagnosis of FSS with proportionate phenotype, thereby expanding the clinical heterogeneity of *FGFR3* N540S variants. These findings underscore the value of genetic testing in ISS, enabling definitive etiologic diagnosis, surveillance of potentially affected organ systems, early therapeutic intervention, and prenatal counseling for at-risk pregnancies. Given the proband’s young age, systematic longitudinal surveillance for HCH-associated manifestations remains ongoing.

All three affected individuals harbored the *FGFR3* N540S variant, classified as a likely pathogenic variant by ACMG criteria and previously reported as pathogenic or likely pathogenic. Structurally, the FGFR3 protein comprises extracellular immunoglobulin-like domains (I, II, III), a transmembrane domain (TM), and intracellular tyrosine kinase domains (TK1 and TK2) (15). Variants within the TK domain, such as K650E (TK2), activate FGFR3 by mimicking ligand-mediated receptor dimerization and the consequent conformational changes that normally induce autophosphorylation (16). The N540 residue resides within the TK1 domain. Notably, serine (S), threonine (T), and tyrosine (Y) represent common phosphorylation sites (17); the substitution of asparagine (N) by serine (S) at this position may enhance phosphorylation potential, thereby augmenting FGFR3 activation. The absence of skeletal manifestations in this pedigree may reflect differential inhibitory effects on osteochondral proliferation among distinct variants. Prior functional studies have established a hierarchy of growth plate inhibition: wild-type < N540K (HCH) < G380R (ACH) ≤ R248C (TD I) = Y373C (TD I) < K650M (TD I) ≤ K650E (5). The N540S variant, situated at the identical codon as N540K, demonstrates lower phosphorylation activity than N540K (18), potentially accounting for the attenuated phenotypic expression. Although N540S has been predominantly reported in HCH, its association with proportionate short stature has not been previously described. This inaugural report of FSS further elucidates genotype-phenotype correlations and warrants heightened vigilance for *FGFR3* N540S variants in patients with proportionate short stature, particularly those with severe familial short stature.

GH therapy has demonstrated efficacy in improving linear growth in both FSS and HCH. In July 2003, the U.S. Food and Drug Administration (FDA) approved GH for ISS, specifically targeting prepubertal children with height SDS between −2 and −3 or below (19). Since 1997, GH therapy has been approved in Japan for skeletal dysplasias, including ACH and HCH (20). Multiple studies have documented that GH not only augments linear growth but also favorably modifies body proportions, with the most pronounced effects observed during the first year of treatment; however, data regarding final adult height remain limited (21, 22), necessitating further clinical evidence. An alternative targeted therapeutic agent for HCH—vosoritide, has been shown to sustainably ameliorate height deficits with a favorable safety profile, without exacerbating disproportionate body ratios (23). Nevertheless, vosoritide is currently approved only for ACH in the United States and the European Union. Since 2022, vosoritide has been administered for ACH under special access programs in Beijing, Shanghai, and Hainan, though its clinical utility continues to be hampered by prohibitive costs and restricted availability. In the present case, the proband’s clinical and radiographic profile supported a diagnosis of proportionate FSS secondary to *FGFR3* variant. Following comprehensive family counseling, rhGH therapy was initiated. At 9 months of treatment, metabolic surveillance—including thyroid function, glycated hemoglobin, and insulin-like growth factor-1—remained within normal limits, with no adverse events observed. Notably, his height improved by 1.18 SD at the most recent evaluation, providing clinical evidence supporting GH therapy in FGFR3 -related short stature.

This study reports the first case of proportionate short stature associated with the *FGFR3* N540S variant. The familial cosegregation pattern further supports the pathogenicity of this locus and expands the phenotypic spectrum of this variant. Our findings reaffirm that distinct variants at different residues—and even different amino acid substitutions at the same codon—can yield divergent phenotypic outcomes. Genetic interpretation must therefore be grounded in clinical context. This study also underscores the necessity of incorporating *FGFR3* genetic testing into the diagnostic algorithm for idiopathic short stature. The Chinese ISS guidelines actively endorse molecular genetic testing to facilitate precision diagnosis and individualized management. Furthermore, the favorable initial response to growth hormone therapy in this case provides preliminary evidence to support this treatment approach in *FGFR3* -related proportionate short stature.

## Data Availability

The original contributions presented in the study are included in the article/supplementary material. Further inquiries can be directed to the corresponding author.
